# Assessment of post-COVID-19 fatigue among female survivors 2 years after hospital discharge: a nested case–control study

**DOI:** 10.1186/s12889-023-17382-0

**Published:** 2023-12-07

**Authors:** Yidan Ye, Chuyue Xiong, Yang Dai, Yan Wang, Xinyue Yang, Lixia Cheng, Chao Hou, Naifu Nie, Huan Tang, Xiangyu Ma, Anqiang Zhang, Guoqiang Cao, Yong He, Ji Jiang, Li Li

**Affiliations:** 1 Department of Respiratory Medicine, Daping Hospital, Third Military Medical University, Army Medical University, Chongqing, 400042 China; 2 Department of Medical and Research Management, Daping Hospital, Third Military Medical University (Army Medical University), Chongqing, China; 3 Wuhan Taikang Tongji Hospital, Wuhan, China; 4 Department of Epidemiology, College of Preventive Medicine, Third Military Medical University (Army Medical University), Chongqing, China; 5 Department of Trauma Medical Center, Daping Hospital, State Key Laboratory of Trauma, Burns, and Combined Injury, Third Military Medical University (Army Medical University), Chongqing, China; 6 Wuhan Huoshenshan Hospital, Wuhan, China

**Keywords:** COVID-19, Fatigue, Female, HADS, SARS-CoV-2

## Abstract

**Background:**

Fatigue is a common symptom of long COVID syndrome. Compared to male survivors, females have a higher incidence of post-COVID fatigue. Therefore, long-term follow-up is necessary to understand which groups of females are more vulnerable to post-COVID fatigue.

**Methods:**

This is a nested case–control study of female COVID-19 survivors who were discharged from two designated hospitals in Wuhan, China in 2020, and received 2-year follow-up from March 1 to April 6, 2022. All patients completed the Checklist Individual Strength-subscale subjective fatigue (CIS-fatigue), a chronic obstructive pulmonary disease (COPD) assessment test (CAT), and the Hospital Anxiety and Depression Scale (HADS; including the HADS-Anxiety [HADS-A] and the HADS-Depression [HADS-D]). Individuals with CIS-fatigue scores of 27 or higher were classified as cases. The risk factors for fatigue was analysed with multivariable logistic regression analysis.

**Results:**

A total of 899 female COVID-19 survivors were enrolled for analysis, including 47 cases and 852 controls. Compared with controls, cases had higher CAT, HADS-A and HADS-D scores, and showed a higher prevalence of symptoms, including anxiety (cases vs. controls, 44.7% vs. 4.0%, *p* < 0.001), chest tightness (21.2% vs. 2.3%, *p* < 0.001), dyspnoea (19.1% vs. 0.8%, *p* < 0.001) and so on. In multivariable logistic regression analysis, age (OR, 1.03; 95% CI, 1.01–1.06; *p* = 0.02) and cerebrovascular disease (OR, 11.32; 95% CI, 2.87–43.00; *p* < 0.001) were risk factors for fatigue. Fatigue had a statistically significant moderate correlation with depression (*r* = 0.44, *p* < 0.001), but not with CAT ≥ 10.

**Conclusion:**

Female COVID-19 patients who had cerebrovascular disease and older age have higher risk of fatigue. Patients with fatigue have higher CAT scores, and are more likely to have concurrent depression.

**Supplementary Information:**

The online version contains supplementary material available at 10.1186/s12889-023-17382-0.

## Introduction

Coronavirus Disease 2019 (COVID-19) has spread worldwide, with over 767 million confirmed cases as of 19 June 2023. Clinical manifestations include fever, cough, dyspnoea, myalgia, fatigue, normal or decreased leukocyte counts, and radiographic evidence of pneumonia [1]. Therapeutic options for COVID-19 remain limited, with some available in the market showing lower efficacy in real-world settings [2]. As patients recover from the acute phase, persistent, prolonged, and often debilitating sequelae are increasingly recognised in convalescent individuals, named ‘post-COVID-19 syndrome’ or ‘long COVID’ [3–5]. Long COVID was defined by WHO as symptoms in those with SARS-CoV-2 infection, usually 3 months from the onset of COVID-19 and last for at least 2 months and cannot be explained by other diagnoses [6].

Fatigue has been consistently reported as one of the most significant symptoms of long COVID [7]. Previous studies have reported that 20–60% of patients suffer from post-COVID-fatigue [8, 9]. Although all symptoms continued to improve during the follow-up visit, recent studies have shown that fatigue is the most common symptom, regardless of the recovery time [10, 11]. According to a study by Cao et al. [10], sex, education, and preexisting comorbidities were risk factors in patients with post-COVID-19 fatigue who were discharged from hospital. Mazurkiewicz et al. included 303 non-hospitalised patients with COVID-19 and found that females more often suffered from persistent fatigue [12]. Of note, more females suffer from fatigue than males, and current evidence supports that female sex is a risk factor for post-COVID symptoms, including fatigue, anxiety, and depression [13]. However, few studies have explained the risk factors that contribute to the development of fatigue among female and few have quantitatively revealed the correlation between fatigue and mood disorders (including anxiety and depression). Therefore, further clinical analyses focusing on females are necessary to demonstrate which groups of females are more vulnerable to post-COVID fatigue up to 2 years after illness onset. Therefore, this study aimed to investigate the risk factors for fatigue in female COVID-19 survivors and the correlation between fatigue and mood disorders.

## Methods

### Study design and cohort

This study employed a nested case–control design using a multicentred prospective study cohort of COVID-19 patients who were discharged from Huoshenshan Hospital and Taikang Tongji Hospital (both in Wuhan, China) between February 12 and April 10, 2020. One-year and 2-year follow up (from March 1 to April 6, 2022) visits were performed on this cohort to investigate the long-term symptom burden of COVID-19, which have been reported in our previous studies [14]. The study was conducted in accordance with the Strengthening the Reporting of Observational Studies in Epidemiology (STROBE) reporting guidelines for nested case–control studies and was approved by the Ethics Committee of the Daping Hospital, an affiliated hospital of Army Medical University (No. 202153). Informed consent was obtained from all participants or their legal representatives before the survey. All the methods were carried out in accordance with relevant guidelines and regulations from Declaration of Helsinki.

### Case and control definition

The inclusion criterion was that female patients at 2 year follow up who had completed the Checklist Individual Strength–subscale subjective fatigue (CIS-fatigue), a chronic obstructive pulmonary disease (COPD) assessment test (CAT), and the Hospital Anxiety and Depression Scale (HADS; including the HADS-Anxiety [HADS-A] and the HADS-Depression [HADS-D]). The exclusion criteria included (1) those who declined to participate, (2) those unable to be contacted and (3) those deceased. CIS-fatigue, a standardized questionnaire with high internal consistency and test–retest reliability, is used to assess fatigue symptoms. The questionnaire consists of 8 items scored on a 7-point Likert scale. Total Scores range from 8 to 56 points, and a higher score indicates more clinical symptoms of general fatigue. Individuals with CIS-fatigue scores of 27 or higher were classified as cases, while those with scores below 27 were classified as controls.

### Procedures and data acquisition

All female patients were contacted in the order of their discharge date documented in their medical records, and were interviewed via telephone by trained physicians. Various questionnaires were used, including a self-reported symptom table, CIS-fatigue [15, 16], CAT, and HADS (including HADS-A and HADS-D) (Additional file 1). CAT, initially designed to assess symptom burden of patients with COPD [17], was also capable to be applied to assess symptom burden of COVID-19 survivors [18]. HADS was used to measure mood symptoms of anxiety and depression [19]. Each subscale consists of 7 questions with a 4-point Likert scale (0–3). The scores of at least 8 indicates the presence of symptoms of anxiety or depression [20]. The self-reported symptom questionnaire included sweating, chest tightness, anxiety, myalgia, palpitation, cough, chest pain, dizziness, expectoration, dyspnea, headache, edema, taste change, smell reduction, sore throat, anorexia, diarrhea, hemoptysis, nausea, chill, vomiting, rhinobyon, short of breath, abdominal pain, hearing loss, alopecia, joint, and back pain. In this article, “symptoms-2y” was short for the number of symptoms reported in at 2-year follow-up.

Clinical data of patients during hospitalization were retrieved from electronic medical records, including demographic characteristics (self-report age and sex), clinical characteristics (self-reported comorbidities), and clinical treatments (ICU admission, oxygen therapy, and mechanical ventilation). In this article, "co-burden" was short for the number of self-reported comorbidities.

Disease severity was defined by World Health Organization guideline for COVID-19. Severe pneumonia refers to fever or suspected respiratory infection, plus one of the following: respiratory rate greater than 30 breaths per minute, severe respiratory distress, or oxygen saturation as measured by pulse oximetry (SpO_2_) less than or equal to 93% on room air [21]. We double-entered and validated all data using EpiData software version 3.1 (EpiData Association).

### Statistical analysis

Continuous variables were presented as median (IQR), followed by Mann–Whitney U test, and categorical variables were presented as absolute values along with percentages, followed by the Pearson χ2 test or Fisher exact test when appropriate.

To identify factors associated with the risk of occurrence of fatigue defined by CIS-fatigue, univariable logistic regression analysis was used to identify potential risk factors with *p* < 0.10, and then it was adjusted by a stepwise (forward likelihood ratio) selection process in multivariable logistic regression model. All the scores of scales were subjected to Spearman correlation. Each test was 2-sided, *p* < 0.05 was considered significant, and correlation coefficient > 0.6 was considered highly associated. Data were analysed with SPSS statistical package version 26.0 for Windows (IBM SPSS Statistics) and R statistical software version 4.1.1 (R Project for Statistical Computing).

## Results

### Patient characteristics

**Fig. 1:**
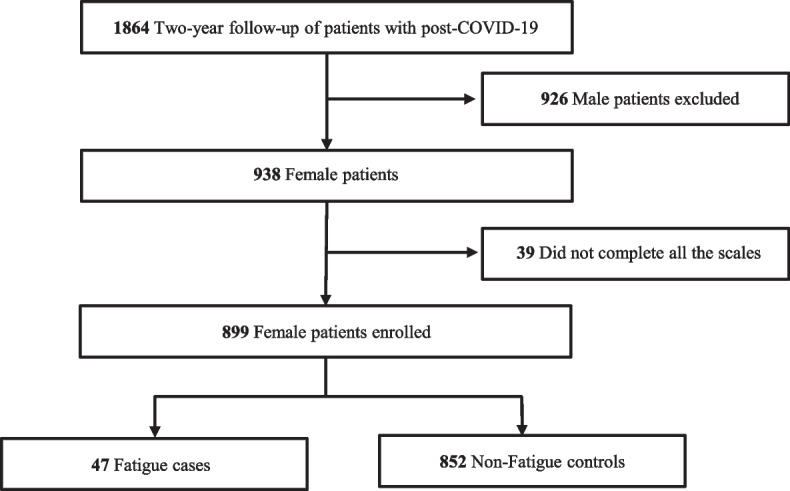
Study flowchart

After 2 years discharged from the hospital, 1864 patients were successfully followed up including 938 female patients. And 899 female patients were enrolled in the study for current analysis, except 39 participants who didn’t finish all the scales. Among them, 47 (5.2%) were classified as cases based on the score of CIS-fatigue (Fig. 1 and Additional file 2), while the remaining 852 were controls. The median (IQR) age of enrolled patients was 59 (50–68), and the median (IQR) length of hospital stay was 14 (8–20) days. During hospitalization, 17 patients (1.9%) were admitted to the ICU, and 627 patients (69.7%) received oxygen therapy (Table 1).

**Table1:** Characteristics of enrolled patients

	Patients, n (%)			*P*-value
Characteristic	Enrolled, *N* = 899^a^	Cases, *N* = 47^a^	Controls, *N* = 852^a^	Cases vs Controls
Age^d^	59 (50, 68)	66 (57, 71)	58 (50, 67)	< 0.001‖
disease severity^b^				0.33
Nonsevere	675 (75.0%)	32 (68.0%)	643 (75.5%)	
severe	224 (24.9%)	15 (31.9%)	209 (24.5%)	
Length of hospital stay^d^	14 (8, 20)	15 (10, 20)	14 (8, 20)	0.62
ICU admission^c^	17 (1.9%)	0 (0.0%)	17 (2.0%)	> 0.99
Oxygen therapy^b^	627 (69.7%)	38 (80.9%)	589 (69.1%)	0.12
Mechanical ventilation^c^	5 (0.6%)	0 (0.0%)	5 (0.6%)	> 0.99
Hypertension^b^	247 (27.4%)	20 (42.6%)	227 (26.6%)	0.027‖
Diabetes^b^	117 (13.0%)	12 (25.5%)	105 (12.3%)	0.017‖
Cardiovascular disease^c^	75 (8.3%)	5 (10.6%)	70 (8.2%)	0.58
Chronic liver disease^c^	26 (2.9%)	3 (6.4%)	23 (2.7%)	0.15
Cerebrovascular disease^c^	11 (1.2%)	5 (10.6%)	6 (0.7%)	< 0.001‖
Chronic kidney disease^c^	19 (2.1%)	2 (4.3%)	17 (2.0%)	0.26
Tumor^c^	15 (1.7%)	1 (2.1%)	14 (1.6%)	0.56
Tracheitis^c^	9 (1.0%)	2 (4.3%)	7 (0.8%)	0.048
COPD^c^	1 (0.1%)	0 (0.0%)	1 (0.1%)	> 0.99
coBurden^d^				< 0.001‖
1	203 (22.6%)	13 (27.7%)	190 (22.3%)	
2	103 (11.5%)	9 (19.1%)	94 (11.0%)	
3	34 (3.8%)	5 (10.6%)	29 (3.4%)	
4	1 (0.1%)	1 (2.1%)	0 (0.0%)	
5	1 (0.1%)	0 (0.0%)	1 (0.1%)	

*COPD* chronic obstructive pulmonary disease

‖significant at α = 0.05

^a^Median (IQR); Frequency (%)

^b^Pearson's Chi-squared test

^c^Fisher's exact test

^d^Wilcoxon rank sum test

The median (IQR) age of cases was 66 (57–71), while the median (IQR) age of controls was 58 (50–67). Compared with controls, cases suffered from higher prevalence of hypertension (cases vs controls, 42.6% vs 26.6%, *p* = 0.027), diabetes (cases vs controls, 25.5% vs 12.3%, *p* = 0.017), cerebrovascular disease (cases vs controls, 10.6% vs 0.7%, *p* < 0.001) and tracheitis (cases vs controls, 4.3% vs 0.8%, *p* = 0.048). However, no significant differences were found in terms of disease severity, the length of hospital stay, and the percentage of other diseases (eg. cardiovascular disease, chronic liver disease, chronic kidney disease or COPD) (Table 1).

### Characteristics of long COVID syndrome at 2-year follow-up

**Fig. 2:**
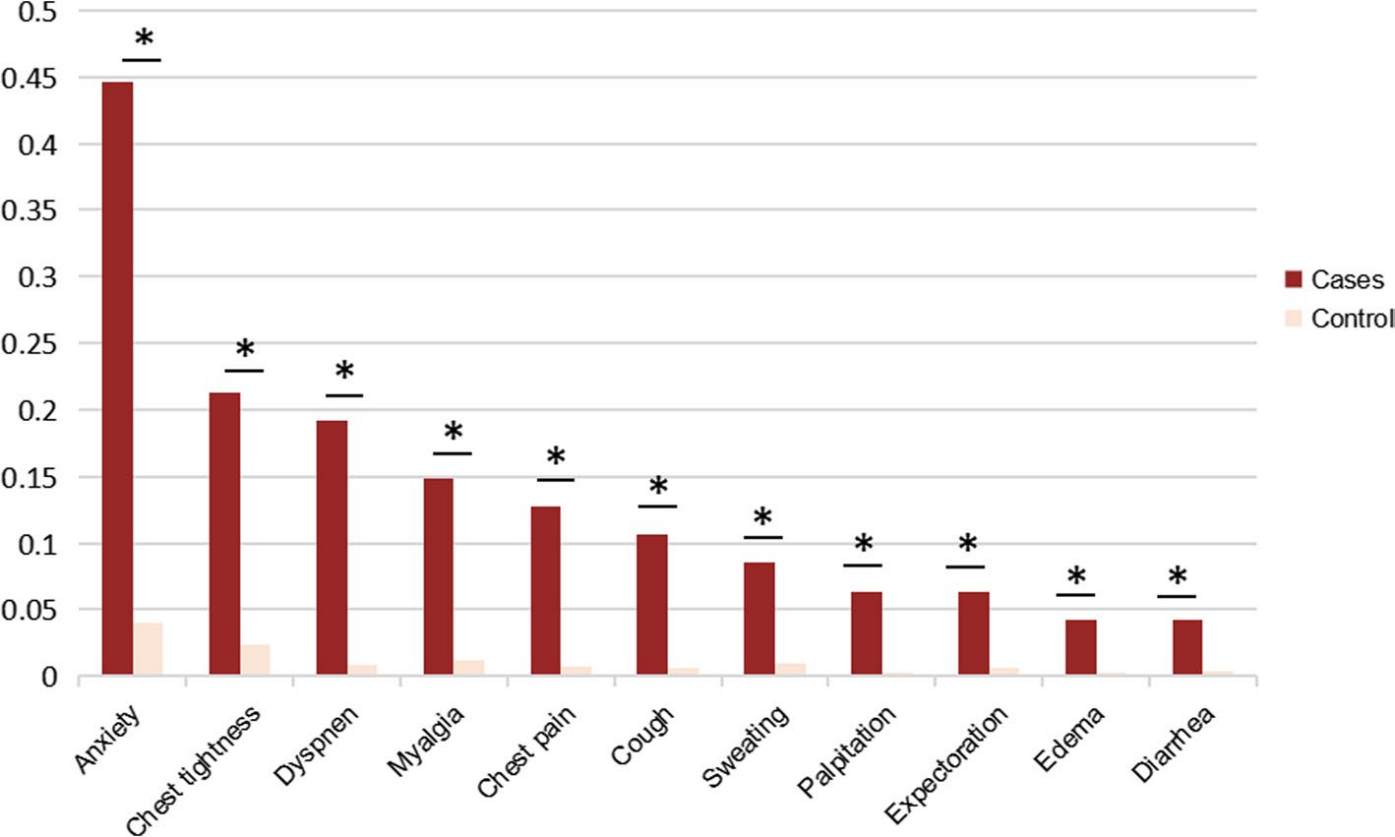
The percentage of symptom burden of cases and controls. *significant at α = 0.05 level

The most common post-COVID symptoms in cases were anxiety (44.7%), chest tightness (21.2%) and dyspnea (19.1%) while in controls were anxiety (4.0%), joint and back pain (3.3%) and chest tightness (2.3%) (Additional file 3). The prevalence of several symptoms was significantly higher in cases compared to controls, such as sweating, chest tightness, anxiety, myalgia, palpitation, cough, chest pain, expectoration, dyspnea, edema and diarrhea (Fig. 2). Besides, those symptoms were subjected to Spearman correlation. In cases, chest pain was highly associated with chest tightness (*r* = 0.74, *p* < 0.001) while expectoration was highly associated with cough (*r* = 0.76, *p* < 0.001) (Additional file 4).

### CAT scores at 2-year follow-up

**Fig. 3:**
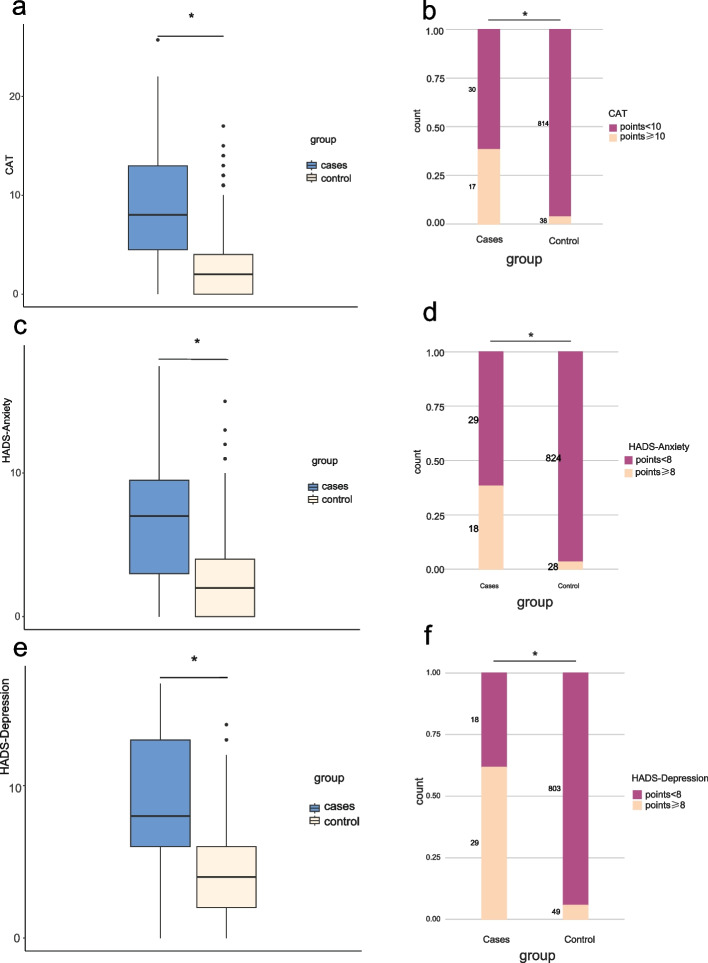
Total scores of CAT, HADS-Anxiety and HADS-Depression in cases and controls. **A** Total CAT score. **B** Percentage of patients with CAT score ≥ 10 and < 10; **C** Total HADS-Anxiety score. **D** Percentage of patients with HADS-A score ≥ 8 and < 8; **E** Total HADS-Depression score. **F** Percentage of patients with HADS-D score ≥ 8 and < 8. *significant at α = 0.05 level

Of the enrolled patients, 55 (6.1%) were grouped as CAT ≥ 10, while 844 (93.8%) were CAT < 10 (Additional file 5). Case group had higher CAT scores than control group (median [IQR], cases vs controls, 8 [4.5–13] vs 2 [0–4]; *p* < 0.001) (Fig. 3a). Among the 47 cases, 17 (36.1%) patients were scored as CAT ≥ 10, and the rate was higher than controls (38[4.5%]; *p* < 0.001) (Fig. 3b).

### HADS-anxiety and HADS-depression scores at 2-year follow-up

A total of 46 (5.1%) patients were grouped as having anxiety (Additional file 6). Cases had higher HADS-anxiety scores than controls (median [IQR], 7 [3–9.5] vs 2 [0–4]; *p* < 0.001) (Fig. 3c). Among cases, 18 (38.3%) were with anxiety, and the rate was higher than that of control subjects (28 [3.3%]; *p* < 0.001) (Fig. 3d).

The overall incidence of depression in survivors was 8.6% (Additional file 7). Cases had higher HADS-depression scores than controls (median [IQR], 8 [6,–13] vs 4 [2,–6]; *p* < 0.001) (Fig. 3e). The rate of depression in cases was 61.7%, which was higher than in controls (49 [5.8%]; *p* < 0.001) (Fig. 3f).

### The risk factors of fatigue at 2-year follow-up

**Fig. 4:**
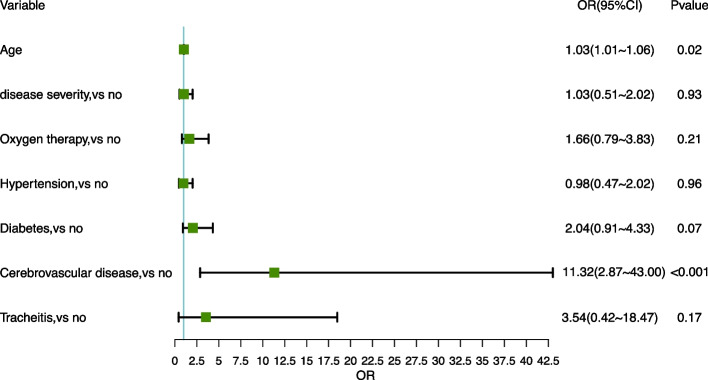
Logistic regression models to evaluate the risk factors for fatigue

**Fig. 5:**
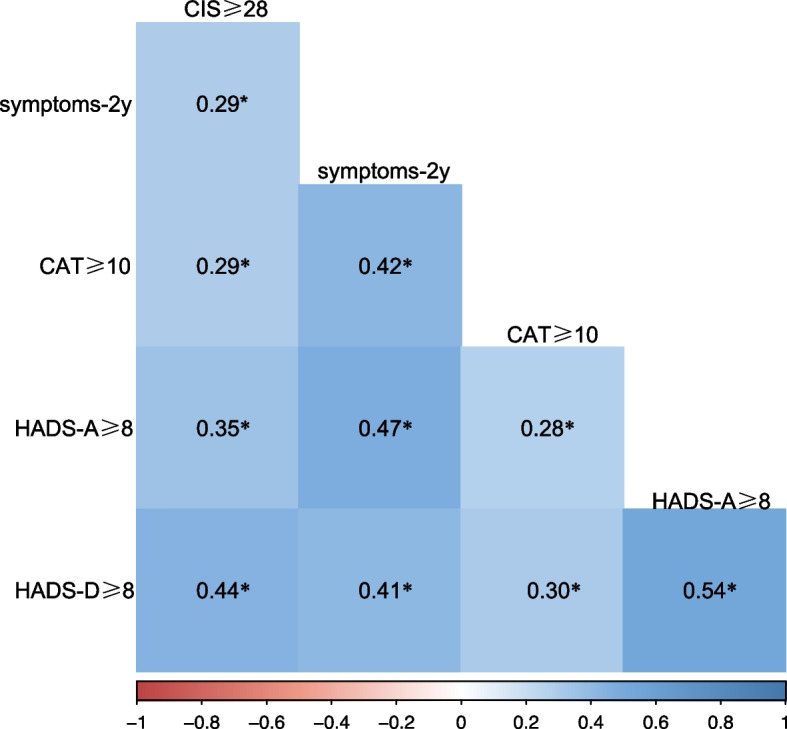
Spearman's rank correlation matrix and correlation significances of relevant variables. *significant at α = 0.05 level

Compared with non-fatigue individuals, age, disease severity, oxygen therapy, hypertension, diabetes, cerebrovascular disease, tracheitis were associated with fatigue under univariable analysis. ln multivariable analysis, age (OR, 1.03; 95%CI, 1.01–1.06; *p* = 0.02) and cerebrovascular disease (OR, 11.32; 95%CI, 2.87–43.00; *p* < 0.001) were the risk factors of fatigue (Fig. 4). Statistically significant moderate correlations were found between CIS ≥ 27 and HADS-D ≥ 8 (*r* = 0.44, *p* < 0.001) (Fig. 5).

## Discussion

This nested case–control study focused on the incidence and risk factors of post-COVID-19 fatigue among female survivors. A significant proportion (5.2%) of survivors still experienced fatigue 2 years after discharge. Compared to controls, those with fatigue demonstrated higher scores on the CAT, HADS-anxiety, and HADS-depression. Age and cerebrovascular disease are risk factors for fatigue. Additionally, there were significant moderate correlations between CIS ≥ 27 and HADS-D ≥ 8. Collectively, these findings add to the current knowledge on post-COVID-19 fatigue, especially among female survivors.

Symptoms contributing to long COVID included fatigue, brain fog, dizziness, palpitations, loss of or change in smell or taste, chronic cough, and chest pain [22]. The underlying pathogenesis of long COVID may conclude three aspects, including immune dysregulation, persistent inflammation, and dysfunction of the endothelium [23]. IL-6, produced by abnormal immunity, may cause pulmonary fibrosis, vascular disease, and psychological disorders [24, 25], even at fatigue onset [26]. Sustained inflammation in the central-peripheral nervous system, which contributes to oxidative stress and autoimmunity, may cause neurocognitive disorders and chronic fatigue [27]. Endothelial dysfunction may lead to inflammation, which is a critical driver of pulmonary vascular diseases and other enduring complications [28].

Fatigue has long been a concern shared by many people, since it is a condition that is not only widely seen after recovery from numerous diseases [29, 30] but is also a typical symptom of patients who have been infected with coronaviruses such as SARS-CoV-1 and MERS [31]. 40.3% of survivors suffered from SARS reported fatigue four years later [32].

For SARS-CoV-2, which is also a coronavirus, fatigue was found to be a common symptom from the outset, with 69% of patients reporting persistent fatigue for nearly 2 months after discharge [33]. Moreover, fatigue is more common in female patients than male. Tracking female patients would help investigate the risk factors for post-COVID-19 fatigue, offer new insights into the sequelae of coronavirus infection, and deepen the understanding of fatigue itself [34]. Promisingly, effective therapies such as pulmonary rehabilitation, exercise training, education, and behavioural changes [5], can improve fatigue in COVID-19 survivors [35].

In the current study, we found that depression, rather than anxiety, was correlated with the onset of fatigue. This may be because, unlike anxiety, the development of depression and fatigue share common pathophysiological mechanisms of immune-mediated injury and neuroinflammation, with their causal relationship already being verified [36]. Age, as an important risk factor for adverse health outcomes (including disease severity, mortality, and severity of sequelae) of COVID-19, is the risk factor for the onset of fatigue in females [37]. There is also evidence that older patients are more likely to develop post-COVID fatigue [38]. Co-occurring cerebrovascular diseases are also risk factors for fatigue. Patients admitted with stroke during the COVID-19 pandemic had a significantly higher probability of death [39], which is one of the risk factors for the severity of COVID-19 [40]. In our study, cerebrovascular diseases also had an impact on the long-term outcomes of COVID-19. Furthermore, post-stroke fatigue is one of the most common complications of stroke. In different regions of the world, female sex is a risk factor for post-stroke fatigue [41], indicating that there may be special connections between female sex and the occurrence of fatigue. What's more, patients with cerebrovascular diseases are more likely to experience chronic fatigue [42].

This study has some limitations. First, the sample size of the study is limited. Because the enrolled patients were less than half of the eligible population discharged from hospital, the potential risk for fatigue may be underestimated. Furthermore, the study assessed physical fatigue but not chronic fatigue syndrome (CFS). CFS is a clinically defined condition characterised by severe disabling fatigue and a combination of symptoms [43]. In previous reports, many symptoms of COVID-19 were similar to those of CFS, thus some researchers have used the CIS-fatigue to evaluate fatigue in patients [16, 44]. Thirdly, patients included were infected with wild-type strain and received hospital admission. The constantly emerging coronavirus variants and the tendcy of non-hospitalized therapy may lead to different health outcomes and risk factors with our findings.

## Conclusion

This study found in female COVID-19 patients, cerebrovascular disease and older age could contribute to a higher risk of fatigue. Patients with fatigue have higher CAT scores, and are more likely to have concurrent depression. More importantly, our findings improved the understanding of the possible causes of fatigue in female survivors in order to develop effective strategies for prevention.

### Supplementary Information

**Additional file 1.** Covid-19 Survivors two-year Clinical Sequelae Follow-up Questionnaire. Scores Distribution on CIS-fatigue. Symptoms of Long Covid Syndrome. Spearman's rank correlation matrix and correlation significances of 11 self-reported symptoms. Scores Distribution on CAT. Scores Distribution on HADS-A. Scores Distribution on HADS-D.

## Data Availability

Restrictions apply to the availability of these data and so they are not publicly available. However, data are available from the corresponding author upon reasonable request and with the permission of the institution.

## Abbreviations

CIS-fatigue: The Checklist Individual Strength-subscale subjective fatigue
COPD: Chronic obstructive pulmonary disease
CAT: A chronic obstructive pulmonary disease (COPD) assessment test
HADS: The Hospital Anxiety and Depression Scale
HADS-A: The HADS-Anxiety
HADS-D: The HADS-Depression
symptoms-2y: The number of symptoms reported in at 2-year follow-up
co-burden: The number of self-reported comorbidities
CFS: Chronic fatigue syndrome
