# Supplementary material for: Assessment of post-COVID-19 fatigue among female survivors 2 years after hospital discharge: a nested case–control study
Source: BMC Public Health. 2023 Dec 7;23:2455. doi: 10.1186/s12889-023-17382-0 (PMC10704782; doi:10.1186/s12889-023-17382-0)
Supplement: Supplementary file 1 — Additional file 1. Covid-19 Survivors two-year Clinical Sequelae Follow-up Questionnaire. Scores Distribution on CIS-fatigue. Symptoms of Long Covid Syndrome. Spearman's rank correlation matrix and correlation significances of 11 self-reported symptoms. Scores Distribution on CAT. Scores Distribution on HADS-A. Scores Distribution on HADS-D. [file 12889_2023_17382_MOESM1_ESM.docx]

**Additional files legends**

**Additional file 1.** Covid-19 Survivors two-year Clinical Sequelae Follow-up Questionnaire

**Additional file 2.** Scores Distribution on CIS-fatigue

**Additional file 3.** Symptoms of Long Covid Syndrome

**Additional file 4.** Spearman's rank correlation matrix and correlation significances of 11 self-reported symptoms

**Additional file 5.** Scores Distribution on CAT

**Additional file 6.** Scores Distribution on HADS-A

**Additional file 7.** Scores Distribution on HADS-D

**Additional file 1.**

**Covid-19 Survivors two-year Clinical Sequelae Follow-up Questionnaire**

Number： Willingness to follow up：□yes □no □lost-to-follow up

1.Follow-up of COVID-19-related symptoms

Are the following symptoms currently present ? How the degree?

| 1.Dyspnea：□no □yes |
| --- |
| 2.Cough：□no □yes |
| 3.Expectoration：□no □yes |
| 4.Rhinobyon：□no □yes |
| 5.Edema：□no □yes |
| 6.Chest tightness：□no □yes |
| 7.Palpitation：□no □yes |
| 8.Dizzness：□no □yes |
| 9.Headache：□no □yes |
| 10.Myalgia：□no □yes |
| 11.Anxiety：□no □yes |
| 12.Sweating：□no □yes |
| 13.Smell reduction：□no □yes |
| 14.Diarrhea：□no □yes |
| 15.Nausea：□no □yes |
| 16.Vomiting：□no □yes |
| 17.Chest Pain：□no □yes |
| 18.Abdominal pain：□no □yes |
| 19.Hearing loss：□no □yes |
| 20.Alopecia：□no □yes |
| 21.Back pain：□no □yes |
| 22.Joint pain：□no □yes |
| Other COVID-19-related symptoms, if any: |

2.CAT scoring Total score：______

| Symptoms | score | Symptoms |
| --- | --- | --- |
| I never cough | 0 1 2 3 4 5 | I cough all the time |
| I have no phlegm (mucus) in my chest at all | 0 1 2 3 4 5 | My chest is completely full of phlegm (mucus) |
| My chest does not feel tight at all | 0 1 2 3 4 5 | My chest feels very tight |
| When I walk up a hill or one flight of stairs I am not breathless | 0 1 2 3 4 5 | When I walk up a hill or one flight of stairs I am very breathless |
| I am not limited doing any activities at home | 0 1 2 3 4 5 | I am very limited doing activities at home |
| I am confident leaving my home despite my lung condition | 0 1 2 3 4 5 | I am not at all confident leaving my home because of my lung condition |
| I sleep soundly | 0 1 2 3 4 5 | I don’t sleep soundly because of my lung condition |
| I have lots of energy | 0 1 2 3 4 5 | I have no energy at all |

3.CIS fatigue subscale Total score：______

| 1.I feel tired  Yes, that is true □7 □6 □5 □4 □3 □2 □1 No, that is not true |
| --- |
| 2.Physically, I feel exhausted  Yes, that is true □7 □6 □5 □4 □3 □2 □1 No, that is not true |
| 3. I feel fit  Yes, that is true □1 □2 □3 □4 □5 □6 □7 No, that is not true |
| 4. I feel weak  Yes, that is true □7 □6 □5 □4 □3 □2 □1 No, that is not true |
| 1. I feel rested   Yes, that is true □1 □2 □3 □4 □5 □6 □7 No, that is not true |
| 1. Physically,I feel I am in bad condition   Yes, that is true □7 □6 □5 □4 □3 □2 □1 No, that is not true |
| 7.I am tired very quickly  Yes, that is true □7 □6 □5 □4 □3 □2 □1 No, that is not true |
| 8.Physically,I feel in good shape  Yes, that is true □1 □2 □3 □4 □5 □6 □7 No, that is not true |

4.HAD

| Statement | Did not apply to me at all | Applied to me to some degree or some of the time | Applied to me a considerable degree or a good part of the time | Applied to me very much or most of the time |
| --- | --- | --- | --- | --- |
| 1.I feel tense or wound up | 0 | 1 | 2 | 3 |
| 2.I get a sort of frightened feeling as if something bad is about to happen | 0 | 1 | 2 | 3 |
| 3. Worrying thoughts go through my mind | 0 | 1 | 2 | 3 |
| 4.I can sit at ease and feel relaxed | 0 | 1 | 2 | 3 |
| 5.I get a sort of frightened feeling like butterflies in the stomach | 0 | 1 | 2 | 3 |
| 6.I feel restless and have to be on the move | 0 | 1 | 2 | 3 |
| 7.I get sudden feelings of panic | 0 | 1 | 2 | 3 |
| Total score(Anxiety)： |  | | | |
| 1.I still enjoy the things I used to enjoy | 0 | 1 | 2 | 3 |
| 2.I can laugh and see the funny side of things | 0 | 1 | 2 | 3 |
| 3.I feel cheerful | 0 | 1 | 2 | 3 |
| 4.I feel as if I am slowed down | 0 | 1 | 2 | 3 |
| 5.I have lost interest in my appearance | 0 | 1 | 2 | 3 |
| 6.I look forward with enjoyment to things | 0 | 1 | 2 | 3 |
| 7.I can enjoy a good book or radio or TV program | 0 | 1 | 2 | 3 |
| Total score(depression)： |  | | | |

**Additional file 2.**

**
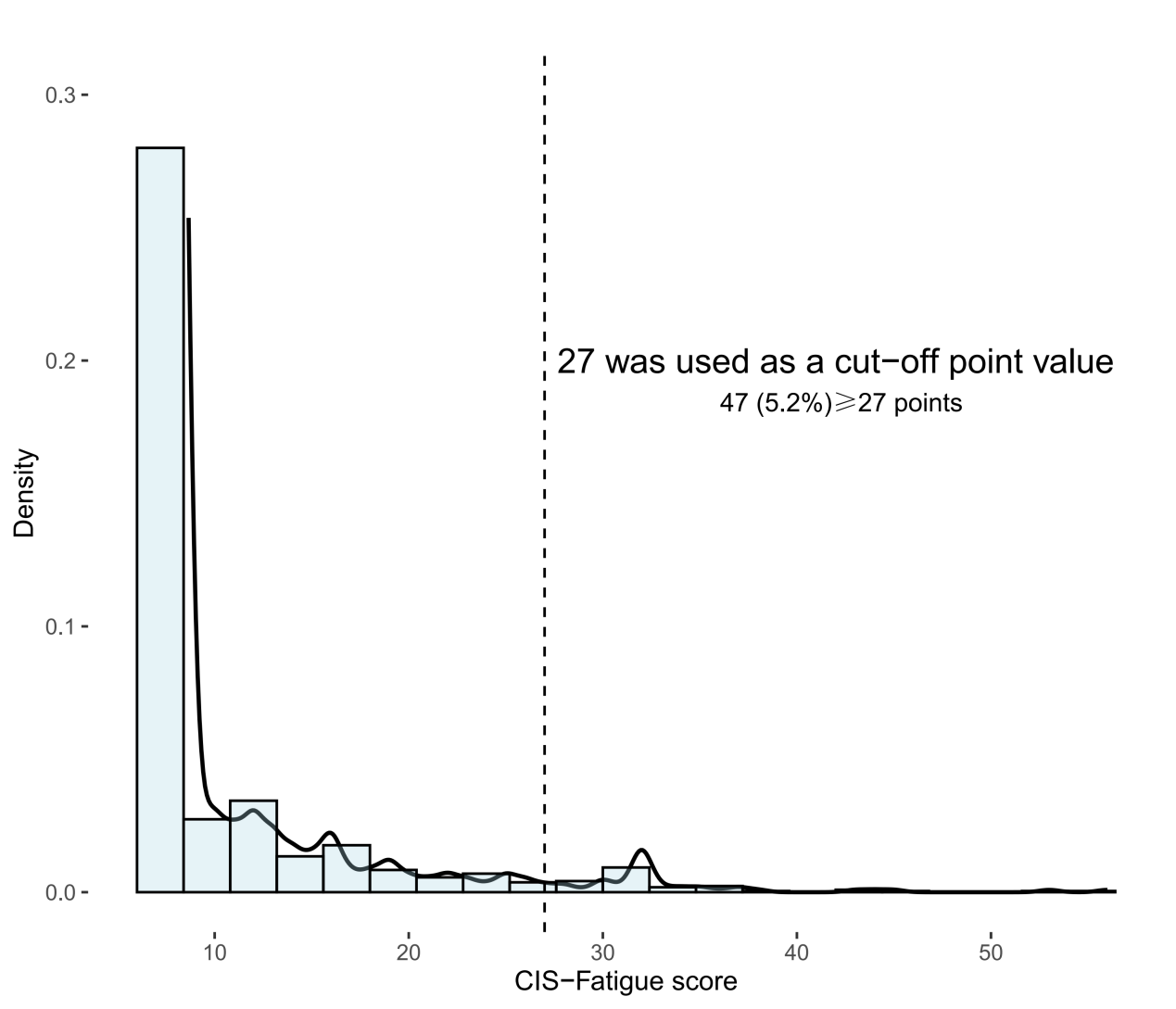
**

**Additional file 3.**

|  | Patients, n (%) | |  | | | *P*-value |
| --- | --- | --- | --- | --- | --- | --- |
| Characteristic | Cases, N = 47 | Controls, N = 852 |  |  |  | |
| Sweating*† | 4 (8.5%) | 8 (0.9%) |  |  | 0.002§ | |
| Chest tightness *† | 10 (21.2%) | 20 (2.3%) |  |  | <0.001§ | |
| Anxiety*† | 21 (44.7%) | 34 (4.0%) |  |  | <0.001§ | |
| Myalgia*† | 7 (14.9%) | 10 (1.2%) |  |  | <0.001§ | |
| Palpitation*† | 3 (6.4%) | 2 (0.2%) |  |  | 0.001§ | |
| Cough*† | 5 (10.6%) | 5 (0.6%) |  |  | <0.001§ | |
| Chest pain*† | 6 (12.8%) | 6 (0.7%) |  |  | <0.001§ | |
| Dizziness*† | 1 (2.1%) | 2 (0.2%) |  |  | 0.15 | |
| Expectoration*† | 3 (6.4%) | 5 (0.6%) |  |  | 0.006§ | |
| Dyspnea*† | 9 (19.1%) | 7 (0.8%) |  |  | <0.001§ | |
| Headache*† | 1 (2.1%) | 5 (0.6%) |  |  | 0.28 | |
| Edema*† | 2 (4.3%) | 1 (0.1%) |  |  | 0.008§ | |
| Smell reduction*† | 0 (0.0%) | 1 (0.1%) |  |  | >0.99 | |
| Diarrhea*† | 2 (4.3%) | 3 (0.4%) |  |  | 0.024§ | |
| Nausea*† | 1 (2.1%) | 2 (0.2%) |  |  | 0.15 | |
| Vomiting*† | 0 (0.0%) | 1 (0.1%) |  |  | >0.99 | |
| Rhinobyon*† | 1 (2.1%) | 0 (0%) |  |  | 0.052 | |
| Abdominal pain*† | 1 (2.1%) | 0 (0%) |  |  | 0.052 | |
| hearing loss*† | 0 (0.0%) | 1 (0.1%) |  |  | >0.99 | |
| Alopecia*† | 0 (0.0%) | 13 (1.5%) |  |  | >0.99 | |
| Joint and back pain*† | 2 (4.3%) | 28 (3.3%) |  |  | 0.67 | |
| Symptoms*‡ |  |  |  |  | <0.001§ | |
| 0 | 15(31.9%) | 683(80.2%) |  |  |  | |
| 1 | 10(21.3%) | 114(13.4%) |  |  |  | |
| 2 | 2(4.3%) | 40(4.69%) |  |  |  | |
| ≥3 | 20(42.6%) | 15(1.76%) |  |  |  | |
| *Frequency (%); †Fisher's exact test; ‡Pearson's Chi-squared test; §significant at α =0.05 | | | | | | |

**Additional file 4.**

**
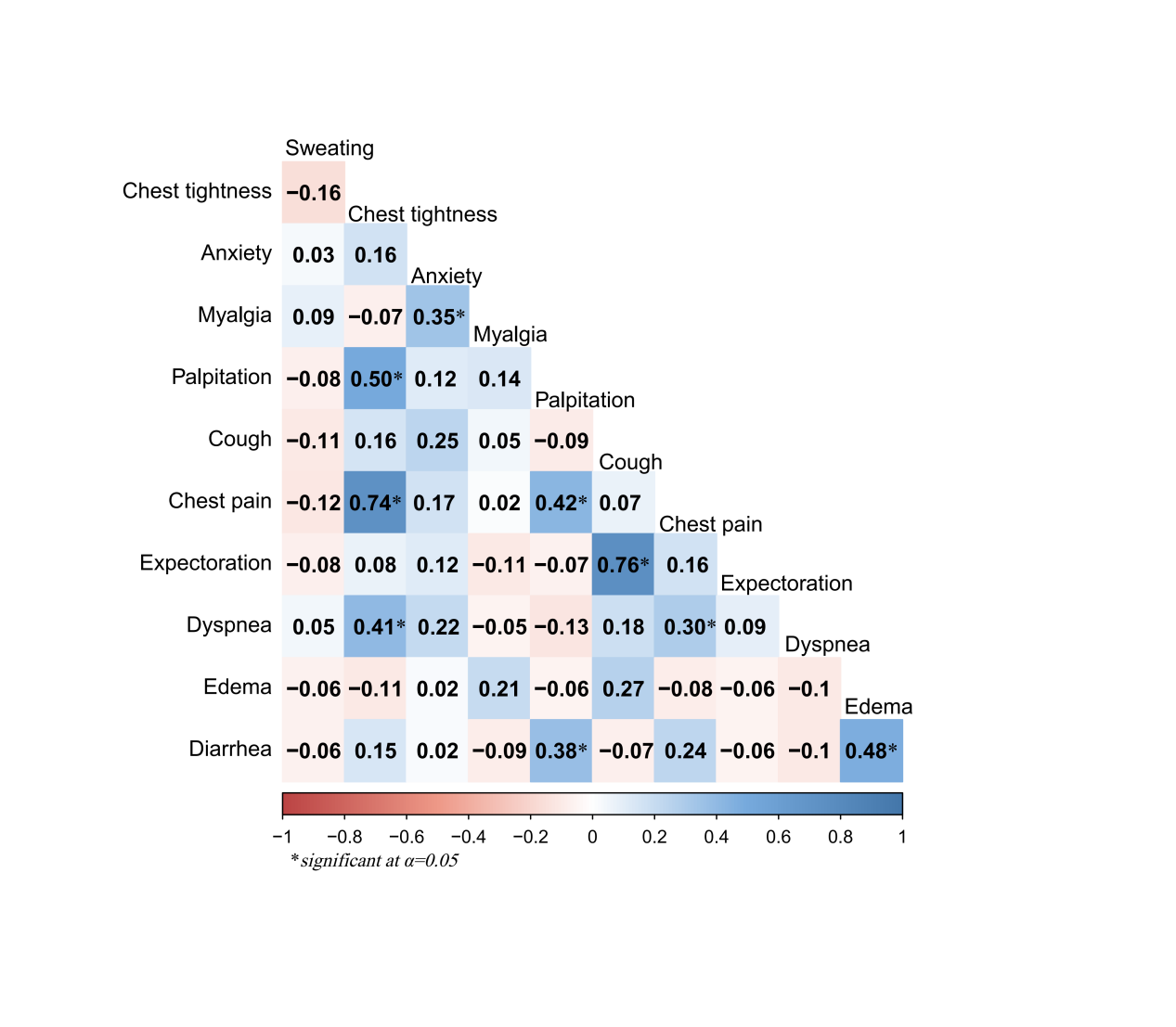
**

**Additional file 5.**

**
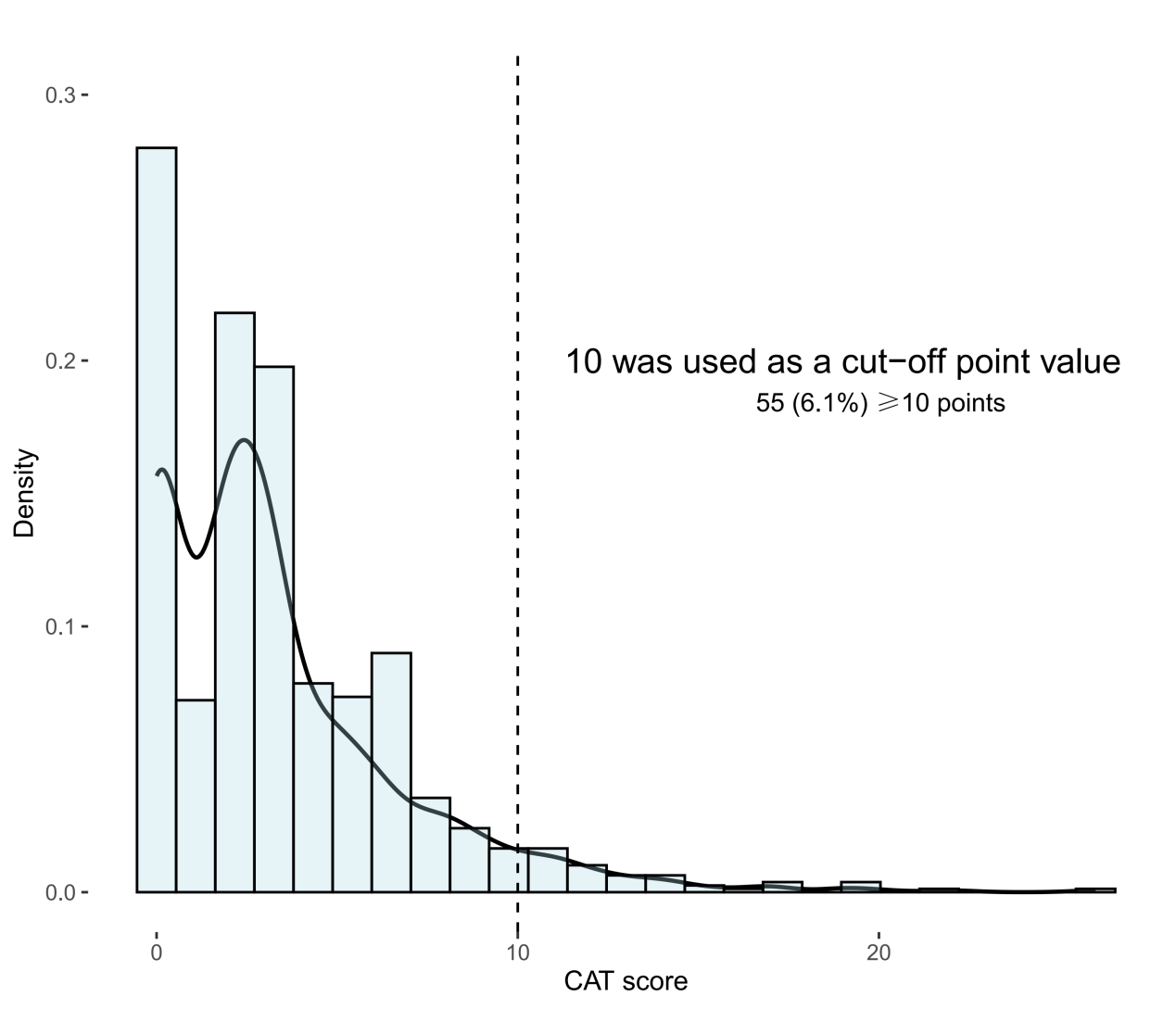
**

**Additional file 6.**

**
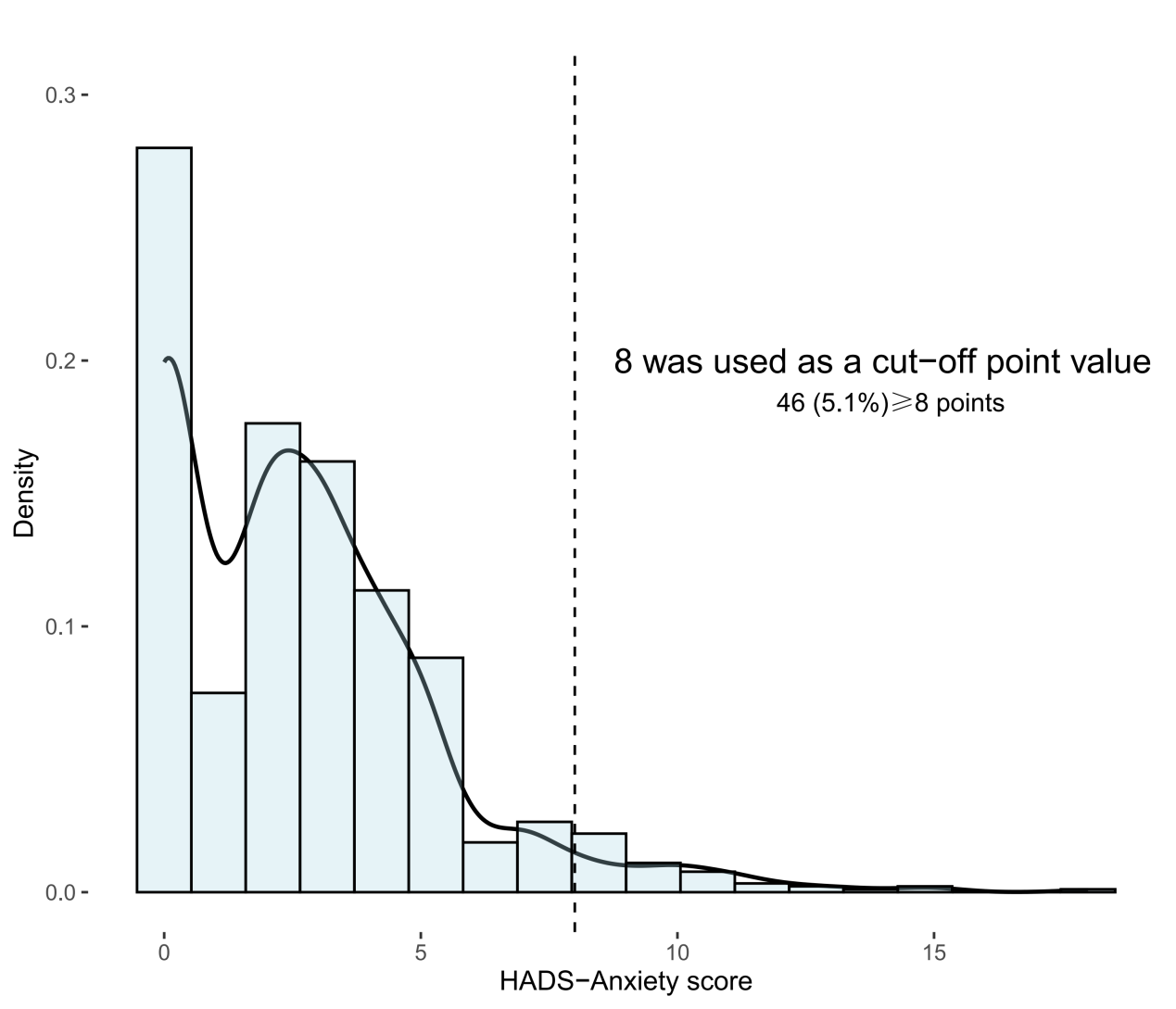
**

**Additional file 7.**

**
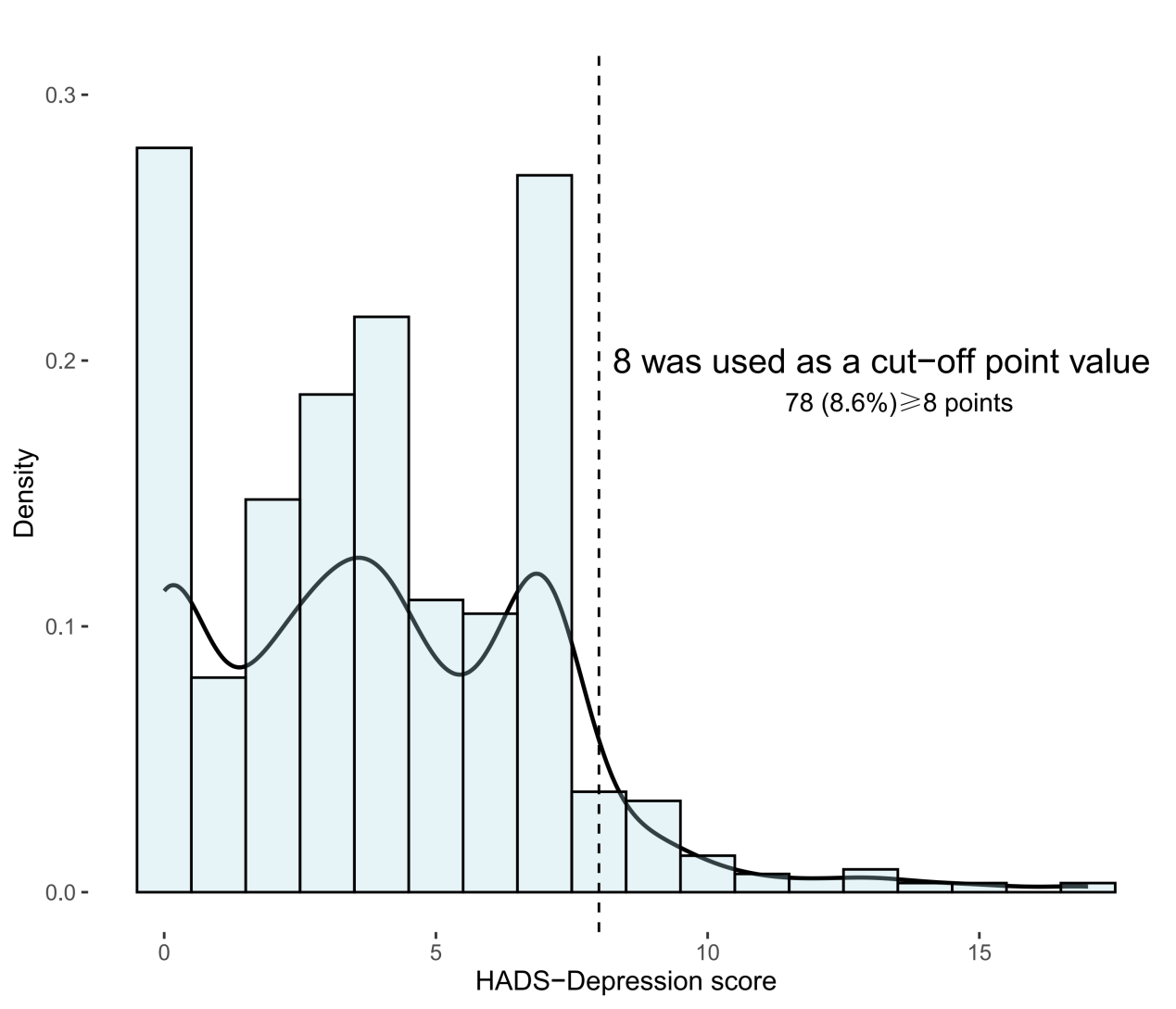
**
